# Exploratory phase II trial in a multicenter setting to evaluate the clinical value of a chemosensitivity test in patients with gastric cancer (JACCRO-GC 04, Kubota memorial trial)

**DOI:** 10.1007/s10120-015-0506-z

**Published:** 2015-09-18

**Authors:** Nobuhiko Tanigawa, Hiroki Yamaue, Shigekazu Ohyama, Shinichi Sakuramoto, Takao Inada, Yasuhiro Kodera, Yuko Kitagawa, Kenji Omura, Masanori Terashima, Yuh Sakata, Atsushi Nashimoto, Toshiharu Yamaguchi, Keisho Chin, Eiji Nomura, San-Woong Lee, Masahiro Takeuchi, Masashi Fujii, Toshifusa Nakajima

**Affiliations:** Department of General and Gastroenterological Surgery, Osaka Medical College, Osaka, Japan; Second Department of Surgery, Wakayama Medical University, Wakayama, Japan; Division of Digestive Surgery, Cancer Center Institute Ariake Hospital, Tokyo, Japan; Departmet of Surgery, Kitasato University, Sagamihara, Tokyo Japan; Division of Surgery, Tochigi Cancer Center, Utsunomiya, Japan; Second Department of Surgery, Nagoya University Graduate School of Medicine, Nagoya, Japan; Department of Surgery, Faculty of Medicine, Keio University, Tokyo, Japan; Division of Surgery, Ageo Central General Hospital, Ageo, Japan; Division of Surgery, Shizuoka Cancer Center, Shizuoka, Japan; Misawa City Hospital, Aomori, Japan; Division of Surgery, Niigata Cancer Center Hospital, Niigata, Japan; Division of Medical Oncology, Cancer Center Institute Ariake Hospital, Tokyo, Japan; Department of Pharmacology, Kitasato University, Sagamihara, Japan; Department of Surgery, Nihon University of School of Medicine, Tokyo, Japan; The Japan Clinical Cancer Research Organization, Tokyo, Japan; Tanigawa Memorial Hospital, 16-59 Kasuga 1-Cho-Me, Ibaraki, Osaka 567-0031 Japan

**Keywords:** Chemosensitivity test, Relapse-free survival, Appropriate cutoff values, Responder, Nonresponder

## Abstract

**Background:**

Although postoperative adjuvant chemotherapy with S-1, an oral fluoropyrimidine, has become a standard of care for gastric cancer in Japan, nonresponders may suffer from the cost and adverse reactions without clinical benefit. This multicenter exploratory phase II trial was conducted to see whether a chemosensitivity test, the collagen gel droplet embedded culture drug sensitivity test (CD-DST), can adequately select patients for chemotherapy.

**Methods:**

The CD-DST using four different concentrations of 5-fluorouracil was conducted with resected specimens from preregistered patients who underwent gastrectomy with D2 or more extensive lymphadenectomy. Patients who were histopathologically confirmed to have stage II or greater disease without distant metastasis were eligible for final enrollment. All patients underwent protocol-specified adjuvant chemotherapy with S-1. Three-year relapse-free survival was compared between patients determined as sensitive by the CD-DST (responders) and those deemed insensitive (nonresponders). Appropriate cutoff values for in vitro growth inhibition were defined when the hazard ratio for relapse in responders and the log-rank *P* values were at their minimum.

**Results:**

Of the 311 patients enrolled, 14 were ineligible and 27 failed to start the protocol treatment. The CD-DST failed in 64 other patients, and survival analyses were conducted with the remaining 206 patients (39 stage II disease, 155 stage III disease, and 12 stage IV disease). The outcome of patients who were determined to be responders was significantly superior to that of nonresponders regardless of the 5-fluorouracil concentrations, although no differences in clinicopathologic characteristics were observed between the two groups, except for age.

**Conclusions:**

The CD-DST identified those who benefit from adjuvant chemotherapy. It deserves further evaluation in the setting of a prospective randomized trial.

ClinicalTrials.gov identifier: NCT00287755

## Introduction

The outcome of patients with resectable gastric cancer has improved owing to the development of technologies making possible earlier diagnosis, as well as the continued progress in surgical techniques and multidisciplinary treatments. However, the outcome remains unsatisfactory in patients with advanced or recurrent disease. Recently, several anticancer agents have been newly introduced, and have raised hope for an improved outcome after chemotherapy. S-1 (TS-1, Taiho Pharmaceutical, Tokyo, Japan) is an oral anticancer drug that combines tegafur (a prodrug of 5-fluorouracil; 5-FU) with 5-chloro-2,4-dihydropyrimidine (CDHP) and potassium oxonate in a molar ratio of 1:0.4:1. A phase III study comparing surgical treatment alone with surgery plus adjuvant S-1 chemotherapy in patients who underwent curative resection of stage II and stage III gastric cancer (Adjuvant Chemotherapy Trial of TS-1 for Gastric Cancer; ACTS-GC) demonstrated that postoperative adjuvant chemotherapy with S-1 significantly improved survival [1, 2]. However, human tumors of even a similar histopathologic category may have markedly different drug sensitivity profiles [3–6]. In vitro drug sensitivity tests have thus been developed to individualize chemotherapy for cancer patients [7–15]. We hypothesize that personalized therapy guided by adequate chemosensitivity testing may lead to a better outcome than conventional empirical therapy. Since the publication of ACTS-GC, orally administered S-1 has become the standard drug for postoperative adjuvant chemotherapy for gastric cancer in Japan [16]. However, this implies that S-1 is also given to patients whose tumors are not sensitive to 5-FU. To address this problem, we organized a research group designated Gastric Cancer 04 (GC-04), consisting of 32 surgical institutions distributed nationwide, in the Japan Clinical Cancer Research Organization (JACCRO). GC-04 conducted this exploratory phase II trial to evaluate the clinical value of chemosensitivity testing of 5-FU in patients who received S-1 postoperatively. Our main goal was to verify whether survival is better in patients whose tumors are sensitive to 5-FU in vitro than in those insensitive to 5-FU in vitro. The primary end point was relapse-free survival (RFS). Secondary end points included 3-year overall survival (OS) and safety. The study was performed from December 2005 to December 2013.

## Materials and methods

The trial was conducted in accordance with the World Medical Association Declaration of Helsinki and Japanese Ethical Guidelines for Clinical Studies. The protocol was approved by the institutional review board of each participating hospital. Written informed consent was obtained from all patients. All members of the steering committee and the sponsor jointly designed the trial and collected the data, which were managed by the independent JACCRO GC-04 Data Center. The data were analyzed by an independent data and safety monitoring committee.

### Eligibility criteria

The eligibility criteria were as follows: (1) presence of histologically proven stage II, stage IIIA, or stage IIIB gastric cancer, and stage IV gastric cancer with N3 but without hepatic, peritoneal, or distant organ metastasis; (2) treatment by D2 or more extensive lymph node dissection; (3) an age of 20–80 years; (4) no previous treatment for cancer; and (5) adequate organ function (a leukocyte count of at least 4,000/ml; a platelet count of at least 100,000/ml; a total bilirubin level of no more than 1.5 mg/dl, aspartate aminotransferase and alanine aminotransferase levels of no more than 2.5 times the upper limit of the normal range; and a serum creatinine level no greater than the upper limit of the normal range). Tumor stage classification and D classification were in accordance with the Japanese Classification of Gastric Carcinoma (second English edition) [17]. For patients to be included in the final analysis, the in vitro sensitivity of tumor tissue to 5-FU had to be successfully evaluated by chemosensitivity testing.

### Drug sensitivity test

The collagen gel droplet embedded culture drug sensitivity test (CD-DST) was used to assess in vitro sensitivity to 5-FU because it is the only commercially available method distributed as a kit, and various studies have demonstrated its usefulness in evaluating the in vitro chemosensitivity of fresh human tumors [18–24]. The CD-DST was performed as described previously [25, 26].

Briefly, a portion of each resected tumor specimen was excised and thinly sliced. Each sample was treated with dispersion enzyme cocktail EZ (Kurabo Industries, Osaka, Japan). The resulting cell suspension was transferred to collagen-coated flasks (CG-flask; Kurabo Industries) and cultured in preculture medium containing 10 % fetal bovine serum at 37 °C in 5 % CO_2_ overnight. The collagen gel was digested with 0.05 % collagenase (type I; Sigma-Aldrich Japan, Tokyo, Japan) to obtain viable cancer cells. The cancer cell suspension prepared was added to reconstructed type I collagen solution (Cellmatrix type CD; Kurabo Industries) to obtain a final cell density of 1 × 10^5^/ml. Three drops of the collagen–cell mixture (30 µl per droplet) were placed in each well of a six-well plate on ice and allowed to gel at 37 °C in a CO_2_ incubator overnight. Subsequently, the tumor cells in the collagen gel droplet were exposed to 5-FU at concentrations corresponding to the area under the drug concentration–time curve in patients and were incubated for 120 h. The 5-FU concentrations used were 0.2, 0.4, 1.0, and 2.0 µg/ml. After removal of the medium containing 5-FU, each well was rinsed twice with 3 ml of Hanks balanced salt solution each time, overlaid with 4 ml of PCM-2 medium (serum-free medium; Kurabo Industries), and incubated for 7 days. At the end of the incubation, neutral red was added to each well at a final concentration of 50 µg/ml, and the colonies in the collagen gel droplet were fixed in 10 % neutral-buffered formalin, washed with water, air-dried, and quantified by optical density image analysis. In vitro sensitivity was expressed as the *T*/*C* ratio, where *T* is the optical density of the 5-FU-treated samples on day 7 and *C* is the optical density of nontreated controls on day 7. The growth inhibition rate (GIR) was calculated as 1 − *T*/*C*.

In the pilot study using 31 fresh gastric cancers that were provided to the central laboratory in the initial stage of this study, we could not find any significant difference in GIR between 5-FU alone and 5-FU with CDHP, which is included in S-1, i.e., GIR of 57.5 ± 22.5 % and 64.3 ± 17.8 % for 5-FU alone at 1.0 and 2.0 µg/ml, respectively, versus GIR of 58.0 ± 20.3 % and 66.4 ± 20.9 % for 5-FU at 1.0 and 2.0 µg/ml with CDHP, respectively. As a result, in vitro sensitivity to 5-FU was used as a surrogate of in vivo sensitivity to S-1 in this study.

### RNA extraction, complementary DNA synthesis, and real-time quantitative reverse transcription polymerase chain reaction

The effect of S-1 can be modulated by the expression levels of 5-FU-related metabolic enzymes, including dihydropyrimidine dehydrogenese (DPD) [27], thymidine synthetase (TS) [28], thymidine phosphorylasa (TP), and orotate phosphoribosyltransferase (OPRT) [29].

Expression levels of TS, DPD, TP, and OPRT messenger RNA (mRNA) were measured as previously [30]. Briefly, total RNA of primary gastric cancer cells was extracted using an Isogen kit (Nippon Gene, Tokyo, Japan) according to the manufacturer’s instructions. Complementary DNA was derived from each sample, and target complementary DNA sequences were amplified by quantitative polymerase chain reaction (PCR) using a fluorescence-based real-time detection method [ABI PRISM 7900 sequence detection system (TaqMan); Applied Biosystems, Foster City, CA, USA]. The PCR conditions were 50 °C for 10 s and 95 °C for 10 min, followed by 42 cycles at 95 °C for 15 s and 60 °C for 1 min. TS, DPD, TP, and OPRT mRNA levels were quantified as ratios between two measurements (gene of interest/β-actin).

### Definition of the appropriate cutoff values

Tumors with a GIR equal to the cutoff value or higher were classified as in vitro sensitive (responders), and those with lower GIRs were classified as in vitro insensitive (nonresponders). After a median follow-up time of 3 years, the hazard ratio (HR) for relapse in the responder group as compared with the nonresponder group was calculated by plotting cutoff values of the in vitro GIR from 10 to 90 % with 10 % increments for each of the four different in vitro 5-FU concentrations. Appropriate cutoff values were defined when the HR for relapse and the log-rank *P* value were at their minimum.

### Study design and treatment

**Fig. 1:**
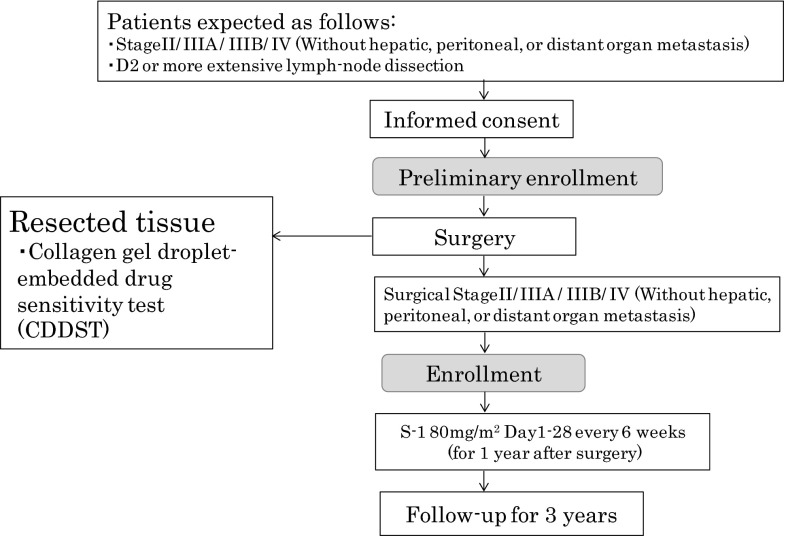
Study schema

Patients were enrolled within 6 weeks after surgery via a Web-based electronic data capture system (FLADS; Takt Systems, Tokyo, Japan) into the JACCRO GC-04 Data Center. Enrolled patients received two oral doses of 40 mg of S-1 per square meter of body-surface area per day for 4 weeks, followed by 2 weeks of no chemotherapy (Fig. 1). During the treatment weeks, the dosages of S-1 were assigned according to body-surface area as follows: less than 1.25 m^2^, 80 mg daily; 1.25 m^2^ or greater to less than 1.5 m^2^, 100 mg daily; and 1.5 m^2^ or greater, 120 mg daily. This 6-week cycle treatment was repeated for 1 year after surgery. If patients had grade 3 or grade 4 hematologic toxicity or grade 2, 3, or 4 nonhematologic toxicity, the daily dose of S-1 was reduced, from 120 to 100 mg, from 100 to 80 mg, or from 80 to 50 mg, respectively.

Patients were followed up for 3 years postoperatively. Adverse events were assessed according to the Common Toxicity Criteria (version 2.0) of the National Cancer Institute.

### Follow-up

Patients underwent hematologic tests and clinical examinations every 2 weeks. Adverse events were evaluated every 3 months for 1 year after surgery.

The presence of recurrence was determined by means of imaging studies, including ultrasonography, computed tomography (CT), gastrointestinal radiography, and endoscopy. Patients underwent at least one type of imaging study, usually CT, at 6-month intervals during the first 2 years after surgery and then at 1-year intervals until 3 years after surgery. Peritoneal relapse was diagnosed when CT or ultrasonography identified cytology-positive ascites. Case-report forms, which included the results of follow-up tests and evaluations and the survival status of patients, were submitted to the JACCRO GC-04 Data Center 0.5, 1, 1.5, 2, and 3 years after surgery.

### Statistical analysis

Our previous retrospective study of 128 patients with gastric cancer demonstrated that survival of the responders as determined by chemosensitivity testing, the histoculture drug-response assay [9], was significantly superior to that of the nonresponders [31, 32]. Taking into consideration the possibilities of failure of chemosensitivity testing and inclusion of ineligible patients, we estimated that a total enrollment of 300 patients would be sufficient to reproduce similar results in the present prospective study. Because the number of days from surgery to enrollment was likely to differ among patients, we decided to calculate the OS and RFS from the date of surgery. All statistical analyses were performed using JMP 8.0 and SAS 9.2 statistical software programs (SAS, Cary, NC, USA). The 3-year RFS and OS rates were estimated using the Kaplan–Meier method, and the log-rank test was used to compare the survival curves. A Cox proportional hazards model was used to calculate the HRs. *P* values less than 0.05 indicated statistical significance.

## Results

### Patients and procedures

**Fig. 2:**
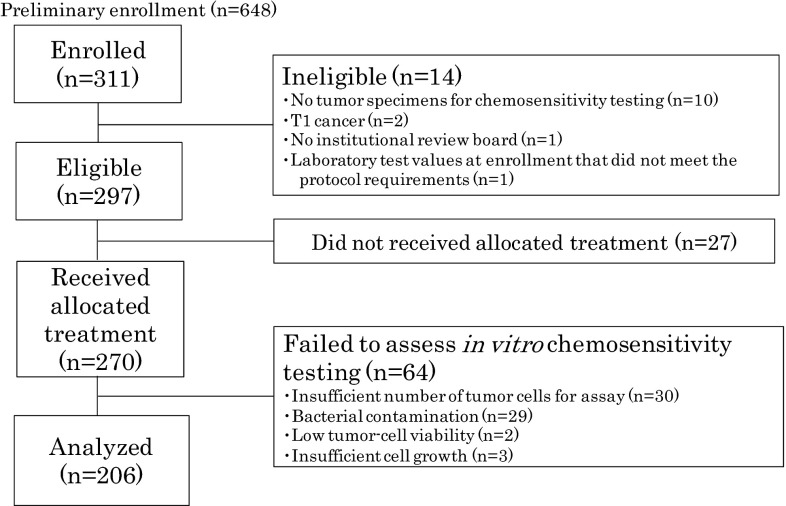
CONSORT diagram

Between December 2005 and December 2013, 311 patients were enrolled at 32 centers in Japan (Fig. 2). At enrollment, 14 patients were found to be ineligible for the following reasons: no tumor specimens available for chemosensitivity testing (ten patients), T1 cancer (two patients), enrollment before approval of the institutional review board (one patient), and laboratory test values at enrollment that did not meet the protocol requirements (one patient). In addition, 27 patients did not receive the protocol treatment of S-1. Among the tumors from the remaining 270 patients, in vitro chemosensitivity was not successfully assessed in 64 tumors for the following reasons: insufficient number of tumor cells for assay (30 patients), bacterial contamination (29 patients), low tumor cell viability (two patients), and insufficient cell growth (three patients). As a result, survival and safety were analyzed in 206 patients in whom chemosensitivity testing was successful.

### Characteristics of the 206 patients

The 206 patients consisted of 151 men and 55 women with a median age of 65 years. Distribution of the disease stage, T stage, N stage, extent of lymph node dissection, ECOG performance status, type of gastrectomy, and tumor histologic type are shown in Table 1.

**Table 1:** Patient characteristics of the 206 patients

Gender	
Male	151 (73.3 %)
Female	55 (26.7 %)
Age	
Median	65 years
Range	32–79 years
Mean	63.5 years
Body surface area (m^2^)	
<1.25	4 (1.9 %)
≥1.25, <1.5	67 (32.5 %)
≥1.5	135 (65.5 %)
Cancer stage^a^	
II	39 (18.9 %)
IIIA	97 (47.1 %)
IIIB	58 (28.2 %)
IV	12 (5.8 %)
T stage^a^	
T1	2 (1.0 %)
T2	52 (25.2 %)
T3	135 (65.5 %)
T4	17 (8.3 %)
N stage^a^	
N0	11 (5.3 %)
N1	109 (52.9 %)
N2	79 (38.3 %)
N3	7 (3.4 %)
Lymph node dissection^a^	
D2	197 (95.6 %)
D3	9 (4.4 %)
ECOG PS	
0	150 (72.8 %)
1	56 (27.2 %)
Type of gastrectomy	
Distal	124 (60.2 %)
Total	82 (39.8 %)
Tumor histology	
Intestinal type	81 (39.3 %)
Diffuse type	123 (59.7 %)
Neuroendocrine cell carcinoma	1 (0.5 %)
Unknown	1 (0.5 %)

*ECOG PS* Eastern Cooperative Oncology Group performance status

^a^Japanese Classification of Gastric Cancer 13th edition

### Adverse events and treatment compliance

Among the 206 patients who received the protocol S-1 treatment, adverse events were evaluated and classified as grade 1, 2, 3, or 4 according to the Common Toxicity Criteria (version 2.0) of the National Cancer Institute. Grade 3 or grade 4 adverse events included neutropenia (10.7 %), diarrhea (5.9 %), mechanical ileus (4.1 %), leukopenia (2.6 %), anorexia (2.6 %), anemia (2.2 %), skin rash (2.2 %), and stomatitis (2.2 %). S-1 treatment was continued for at least 3 months in 183 patients (88.8 %), for at least 6 months in 154 patients (74.8 %), for at least 9 months in 139 patients (67.0 %), and for 12 months in 99 patients (48.1 %). The dose of S-1 was decreased in 76 (36.9 %) of the 206 patients who received the protocol treatment. Of the 99 patients who received the treatment for 12 months, the dose was decreased in 41 patients (41.4 %).

### OS and RFS

On the basis of follow-up data updated as of December 31, 2013, the median follow-up from the time of surgery was 3.2 years in the 206 patients. Forty-seven patients had died. The causes of death were relapse in 39 patients, other cancer in two patients, causes other than cancer in four patients, and unknown causes in two patients. Recurrent diseases occurred in 51 patients. The OS and RFS rates in the 206 patients were 96.1 % and 86.8 %, respectively, at 1 year, 87.7 % and 76.9 %, respectively, at 2 years, and 80.6 % and 71.9 %, respectively, at 3 years.

### Messenger RNA levels of TS, DPD, TP, and OPRT

Gene expression levels of TS, DPD, TP, and OPRT were successfully measured in these 206 tumors. However, mRNA levels of none of these biomarkers correlated with the GIR induced by 5-FU in each of four different 5-FU concentrations (data not shown).

### Association between in vitro sensitivities to 5-FU and survival of patients who received S-1 treatment

**Fig. 3:**
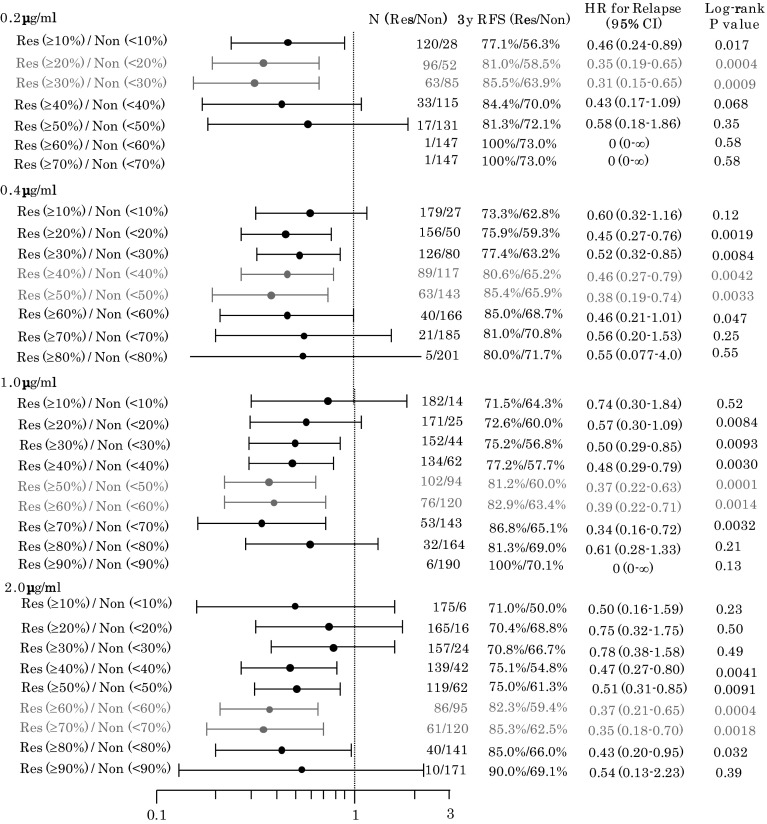
Forest plot to identify appropriate cutoff values for each of the four in vitro 5-fluorouracil concentrations for a total of 206 patients. *CI* confidence interval, *HR* hazard ratio, *Non* nonresponders, *Res* responders, *RFS* relapse-free survival

One of the main purposes of this study was to investigate appropriate cutoff values for classifying patients as likely responders or nonresponders. As described in “Materials and methods,” four different in vitro 5-FU concentrations, which were comparable to clinically achievable plasma 5-FU concentrations, were used to assess the in vitro sensitivity of tumor cells to 5-FU in the CD-DST. The correlation between in vitro chemosensitivity and survival outcome after a 3-year follow-up period is summarized with the forest plot in Fig. 3 in relation to the HR, 3-year RFS, and log-rank *P* value between the responder and nonresponder groups. As shown in Fig. 3, an HR of less than 0.4 with narrow 95 % confidence intervals (CIs) and significant *P* values strongly suggested that appropriate cutoff values for dividing patients into responders and nonresponders were in vitro GIRs of 20–30 % at an in vitro 5-FU concentration of 0.2 µg/ml, 30–40 % at 0.4 µg/ml, 50–60 % at 1.0 µg/ml, and 60–70 % at 2.0 µg/ml. These results indicated that the appropriate cutoff values are substantially influenced by the in vitro drug concentration and can be defined in some, albeit not narrow, ranges.

**Fig. 4:**
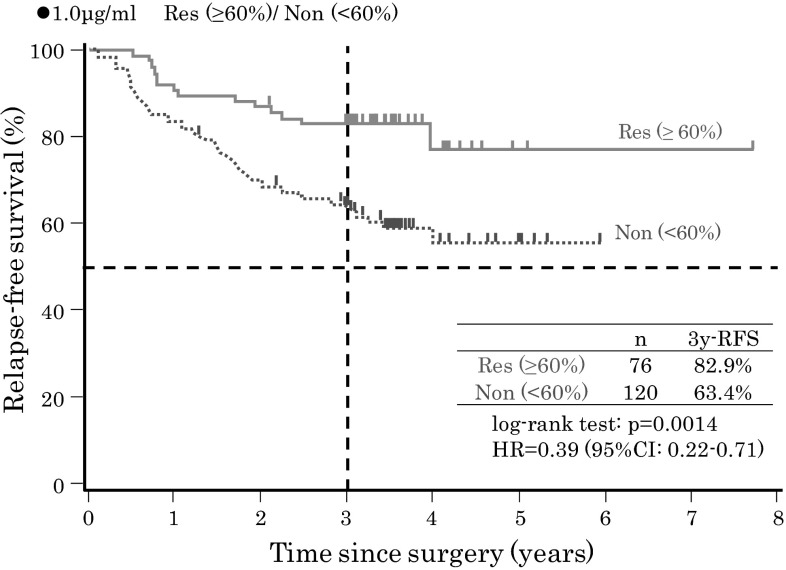
Relapse-free survival (*RFS*) of responder (*Res*) and nonresponder (*Non*) groups classified by a growth inhibition rate of 60 % at an in vitro 5-fluorouracil concentration of 1.0 µg/ml. *CI* confidence interval, *HR* hazard ratio

When these cutoff values were applied, as shown in Fig. 3, responders had significantly better survival than nonresponders for each of the four different in vitro 5-FU concentrations, whereas no significant differences were observed in background clinical characteristics, except for age, between the responder and nonresponder groups. As an example, when an in vitro GIR of 60 % at an in vitro 5-FU concentration of 1.0 µg/ml was applied as a cutoff value, the HR for tumor relapse in the 76 responders, compared with the 120 nonresponders, was 0.39 (95 % CI 0.22–0.71; *P* = 0.0014). The 3-year RFS rate was 82.9 % (95 % CI 74.4–91.3 %) in the responder group and 63.4 % (95 % CI 54.7–72.1 %) in the nonresponder group (Fig. 4), whereas there were no significant differences in background clinical characteristics, except for age, between the responder and nonresponder groups as in Table 2. In addition, as indicated in Table 2, there was no significant difference in relapse sites between the two groups.

**Table 2:** Comparison of background clinical characteristics of responders and nonresponders classified by a growth inhibition rate of 60 % at a 5-fluorouracil concentration of 1.0 µg/ml

Characteristic	Responders (*n* = 76)	Non responders (*n* = 120)	*P*
Gender			0.09
Male	61 (80.3 %)	83 (69.2 %)	
Female	15 (19.7 %)	37 (30.8 %)	
Age	62.5 years (32–78 years)	67.0 years (33–79 years)	0.01
Cancer stage^a^			0.54
II	14 (18.4 %)	23 (19.2 %)	
III	56 (73.7 %)	92 (76.7 %)	
IV	6 (7.9 %)	5 (4.2 %)	
Tumor stage^a^			0.42
T1	0 (0 %)	2 (1.7 %)	
T2	22 (28.9 %)	27 (22.5 %)	
T3	47 (61.8 %)	83 (69.2 %)	
T4	7 (9.2 %)	8 (6.7 %)	
N stage^a^			0.95
N0	4 (5.3 %)	7 (5.8 %)	
N1	38 (50.0 %)	64 (53.3 %)	
N2	31 (40.8 %)	45 (37.5 %)	
N3	3 (3.9 %)	4 (3.3 %)	
Type of lymph node dissection^a^			0.29
D2	71 (93.4 %)	116 (96.7 %)	
D3	5 (6.6 %)	4 (3.3 %)	
ECOG PS			0.18
0	51 (67.1 %)	91 (75.8 %)	
1	25 (32.9 %)	29 (24.2 %)	
RDI^b^	70.2 %	64.3 %	0.29
	(0.9–186 %)	(0.4–119 %)	
Type of gastrectomy			0.80
Total	47 (61.8 %)	72 (60.0 %)	
Distal	29 (38.2 %)	48 (40.0 %)	
Tumor histology			0.54
Intestinal type	27 (35.5 %)	52 (43.3 %)	
Diffuse type	48 (63.2 %)	67 (55.8 %)	
Unknown	1 (1.3 %)	1 (0.8 %)	
Sites of relapse^c^	*n* = 12	*n* = 43	
Local	0 (0 %)	5 (11.6 %)	0.22
Peritoneum	4 (33.3 %)	19 (44.2 %)	0.50
Liver	4 (33.3 %)	11 (25.6 %)	0.59
Distant	4 (33.3 %)	7 (16.3 %)	0.19
Lymph node	4 (33.3 %)	6 (14.0 %)	0.12

There were no significant differences in the background clinical characteristics, except for age, between the responder and nonresponder groups, even when classified by any other defined cutoff values (data not shown)

*ECOG PS* Eastern Cooperative Oncology Group performance status, *RDI* relative dose intensity

^a^Japanese Classification of Gastric Cancer 13th edition

^b^RDI = actual intake of doses/total protocol doses of S-1 for 1 year (%)

^c^Some patients had plural relapses

The HR for tumor relapse of responders compared with nonresponders was 0.24 (95 % CI 0.08–0.68) in 113 patients with N0 or N1 lymph node metastasis and 0.58 (95 % CI 0.25–1.23) in 83 patients with N2 or N3 lymph node metastasis. It was 0.18 (95 % CI 0.00–1.01) in 47 patients with stage II disease and 0.38 (95 % CI 0.18–0.74) in 148 patients with stage III disease.

## Discussion

The CD-DST is a chemosensitivity test wherein isolated tumor cells are embedded in collagen droplets. This three-dimensional culture system has the following advantages over other conventional methods such as 3-(4,5-dimethylthiazol-2-yl)-2,5-diphenyltetrazolium bromide [33] and ATP [34] assays: the ability to use small specimens, the ability to assess the effect of anticancer drugs at physiological concentrations, and the ability to eliminate the masking effect caused by fibroblast contamination in culture with the aid of an image analysis system [26, 35].

The efficacy of the CD-DST in cancer treatment has previously been demonstrated in various malignant human tumors, including gastric cancer [18, 19] and other malignancies [20–24]. However, as recent controversial discussion on chemosensitivity testing for human tumor specimens has indicated [36–42], the studies with the CD-DST also had significant limitations, including small sample sizes, the lack of prospective studies, and the lack of clear cutoff values to distinguish chemotherapy sensitivity from resistance. Accordingly, we conducted this exploratory phase II trial in a multicenter setting to evaluate the clinical value of chemosensitivity testing for 5-FU in patients who received S-1 postoperatively. Our main goal was to verify whether survival is better in patients whose tumors are sensitive to 5-FU in vitro than in those with tumors insensitive to 5-FU in vitro, when appropriate cutoff values to classify the patients as responders and nonresponders were defined.

As one of the major findings of the present study, in vitro chemosensitivity testing of gastric cancer samples using the CD-DST proved to be a feasible method and yielded a success rate of 76 % (206 of 270 samples). Major reasons for unsuccessful assessment of the remaining 64 samples included insufficient number of tumor cells for assay (30 samples) and bacterial contamination (29 samples), as shown in Fig. 2. Both problems may possibly be attributed to the limitations arising from a multicenter setting, such as the inconsistent manner of the handling of tumor samples or the time to transport samples to the laboratory. As a result, there still remains some room for improvement of these technical issues. Also, the test results were obtained within 7 days in all cases, suggesting that the CD-DST may be a useful method in prospective studies to evaluate the clinical significance of sensitivity-test-guided chemotherapy in an adjuvant setting.

As one of accompanying studies in this trial, mRNA expression levels of TS, DPD, TP, and OPRT were quantified by reverse transcription PCR by use of prepared fresh tumor cells. No correlation was found between the mRNA expression of those enzymes and in vitro 5-FU sensitivity, suggesting that it is not possible to predict 5-FU sensitivity solely on the basis of gene expression of the enzymes considered in this study.

Our second finding of this trial was that appropriate cutoff values classifying patients as responders or nonresponders were able to be defined by the calculated HR for tumor relapse and log-rank *P* values. The 3-year RFS rate was significantly better in the responder group than in the nonresponder group when the defined appropriate cutoff values for each in vitro 5-FU concentration were applied. The cutoff values of 50–60 % at a 5-FU concentration of 1.0 µg/ml were already used in previously published reports [18, 19, 21], in which those values were retrospectively determined. Our results verify the finding of the previous studies that there is a direct association between in vitro sensitivity and therapy outcome.

The primary end point of this study was 3-year RFS, the same as in the CLASSIC trial, which was an adjuvant chemotherapy study recently conducted in South Korea [43]. The CLASSIC trial successfully demonstrated a significant survival benefit from adjuvant capecitabine and oxaliplatin chemotherapy compared with surgery alone in patients with stage II–IIIB gastric cancer after D2 surgery. Additionally, in ACTS-GC, whose primary end point was 5-year OS, the 3-year RFS rates were 72.4 and 61.1 % and the 5-year OS rates were 71.7 and 61.1 % in the S-1 group and the surgery-only group, respectively. These findings, in addition to the results of this study, may justify the currently controversial use of the 3-year RFS as the primary end point in clinical trials of adjuvant chemotherapy for potentially curable gastric cancer.

Since the definition of RFS is crucial and very delicate in this set of patients, the follow-up method used was the same as that in ACTS-GC. The absolute number of patients whose relapse was firstly identified was 23 during the first 1 year, 20 between 1 and 2 years, and 8 between 2 and 3 years after surgery in this study, meaning that most of the recurrence occurred within 2 years after surgery. This seems to justify the follow-up method used in the current study.

There were no significant differences between the responder and nonresponder groups in the background clinical characteristics, except for age. The responder group had younger patients than the nonresponder group. However, as also demonstrated in Table 2, the relative dose intensity was almost comparable between these two groups. As a result, the better survival in responders did not seem to be explained by S-1 treatment compliance.

The subset analysis of the HR for tumor relapse of responders compared with nonresponders with respect to tumor stages and lymph node metastases suggested a tendency for a more favorable effect of S1 treatment on patients with an earlier stage of tumor development and of extent of lymph node metastasis, as indicated by ACTS-GC. However, this was not definitely confirmed in this study, probably because of insufficient number of enrolled patients.

In conclusion, to the best of our knowledge, the present phase II study conducted in a multicenter setting is the first large clinical trial to evaluate prospectively the clinical significance of chemosensitivity testing in patients with gastric cancer. Use of the CD-DST may contribute to the proper selection of candidates for chemotherapy and may aid in the reduction of unnecessary adverse events in patients insensitive to 5-FU. This encouraging finding needs further evaluation in a randomized controlled phase III trial to prove that sensitivity-test–guided chemotherapy may provide greater survival benefit than conventional empirical chemotherapy in patients with locally advanced gastric cancer in an adjuvant setting.

## Appendix

Members of the JACCRO GC04 group were as follows: *Steering Committee*: N. Tanigawa, H. Yamaue, S. Sakuramoto, Y. Kodera, Y. Kitagawa, K. Omura, M. Terashima, Y. Sakata, K. Chin, A. Nashimoto, T. Yamaguchi, E. Nomura, M. Takeuchi, M. Fujii, T. Nakajima. *Independent Data and Safety Monitoring Committee*: Y. Shimada, M. Takeuchi, T. Sano. *Medical adviser*: T. Nakajima. *Statistical analysis*: M. Takeuchi. *Principal investigators (all in Japan)*: G. Wakabayashi (Iwate Medical University Hospital), M. Gotoh (Fukushima Medical University Hospital), A. Nashimoto (Niigata Cancer Center Hospital), H. Kuwano (Gunma University Hospital), H. Kato (Dokkyo Medical University Hospital), T. Inada (Tochigi Cancer Center), Y. Yasuda (Jichi Medical University Hospital), S. Sakuramoto (Saitama Medical University International Medical Center), T. Ochiai (Tobu Chiiki Hospital), T. Yamaguchi (Cancer Institute Hospital of JFCR), Y. Kitagawa (Keio University Hospital), Y. Isobe (Tokyo Medical Center), A. Tokunaga (Nippon Medical School Musashi Kosugi Hospital), M. Terashima (Shizuoka Canter Center), T. Yoshikawa (Kanagawa Cancer Center), S. Ito (Aichi Cancer Center Hospital), T. Hara (Kouseiren Takaoka Hospital), K. Yokoi (Kanazawa University Hospital), N. Tanigawa (Osaka Medical College), S. Morita (Hirakata City Hospital), A. Hara (Saiseikai Suita Hospital), K. Hirakawa (Osaka City University Hospital), K. Iwase (Osaka General Medical Center), I. Miyashiro (Osaka Medical Center for Cancer and Cardiovascular Diseases), E. Nakata (Otori Stomachi and Intestine Hospital), T. Nitta (Shiroyama Hospital), J. Matsuyama (Yao Municipal Hospital), H. Yamaue (Wakayama Medical University), K. Yoshikawa (Tokushima University Hospital), Y. Maehara (Kyushu University Hospital), Y. Kitajima (Saga University Hospital), S. Natsugoe (Kagoshima University Hospital).
